# The HPV viral regulatory mechanism of TLRs and the related treatments for HPV-associated cancers

**DOI:** 10.3389/fimmu.2024.1407649

**Published:** 2024-05-15

**Authors:** Shi-Yu Qi, Miao-Miao Yang, Chong-Yang Li, Kun Yu, Shou-Long Deng

**Affiliations:** ^1^ College of Animal Science and Technology, China Agricultural University, Beijing, China; ^2^ College of Chemical and Pharmaceutical Engineering, Jilin Institute of Chemical Technology, Jilin, China; ^3^ Institute of Animal Sciences (IAS), Chinese Academy of Agricultural Sciences (CAAS), Beijing, China; ^4^ National Center of Technology Innovation for animal model, National Health Commission of China (NHC) Key Laboratory of Comparative Medicine, Institute of Laboratory Animal Sciences, Chinese Academy of Medical Sciences and Comparative Medicine Center, Peking Union Medical College, Beijing, China

**Keywords:** human papillomavirus, toll-like receptors, TLR agonists, cancer, TLR therapy

## Abstract

Infection with human papillomavirus (HPV) typically leads to cervical cancer, skin related cancers and many other tumors. HPV is mainly responsible for evading immune tumor monitoring in HPV related cancers. Toll like receptors (TLRs) are particular pattern recognition molecules. When the body is facing immune danger, it can lead to innate and direct adaptive immunity. TLR plays an important role in initiating antiviral immune responses. HPV can affect the expression level of TLR and interfere with TLR related signaling pathways, resulting in sustained viral infection and even carcinogenesis. This paper introduces the HPV virus and HPV related cancers. We discussed the present comprehension of TLR, its expression and signaling, as well as its role in HPV infection. We also provided a detailed introduction to immunotherapy methods for HPV related diseases based on TLR agonists. This will provide insights into methods that support the therapeutic method of HPV related conditions with TLR agonists

## Graphical Abstract

The figure shows the infection factors of HPV, HPV related cancers, and related treatment plans. There are multiple behaviors and factors that can cause HPV infection, including early initiation of sexual activity, multiple sexual partners, childbearing age is young or has multiple pregnancies, long term smoking, long term oral contraceptive pills and weak immunity. Long-term HPV infection can lead to the formation of various cancers, including head and neck cancer, cervical cancer, vaginal cancer, anal cancer and skin cancer. There are currently multiple methods available for preventing and treating related cancers, including therapeutic vaccines, T-cell therapy, natural compounds, nucleic acids based therapy, TLR agonists, checkpoint inhibitor monoclonal antibody.

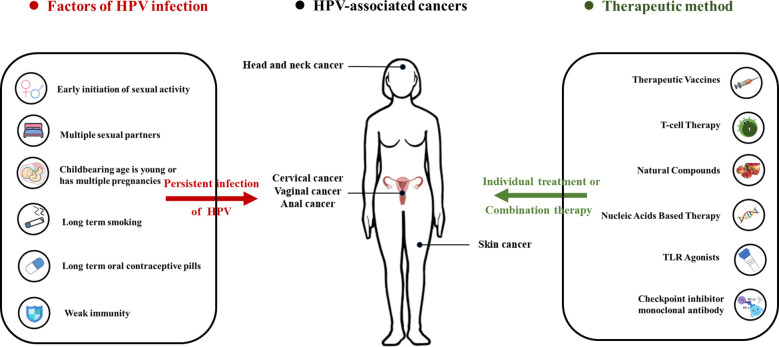

## 1 Introduction

Human Papillomavirus (HPV) is a circular double stranded DNA virus, consisting of over 200 genotypes (1). It is transmitted through contact between the skin or mucous membranes, and gets into the human body through mucosal trauma or skin (2). Usually, some sexually transmitted diseases can cause HPV infection, but in most cases, it can be cleared or cured by the body’s immune system (3). The role of HPV in cancer development has been widely studied, and HPV is an epithelial and species-specific DNA virus. HPV is consisting of low-risk HPV and high-risk HPV. Low-risk HPV mainly causes benign squamous epithelial lesions. The high-risk type can cause the formation of malignant tumors, such as cervical cancer, skin tumors caused by the development of verrucous epidermal dysplasia, cutaneous squamous-cell carcinoma (CSCC), and oropharyngeal squamous cell carcinoma (OSCC) (4). Type 16 and 18 are the most frequent types of HPV. They are also the essential types associated with carcinogenic effects. Both can be prevented by vaccination (3).

**Figure 1:**
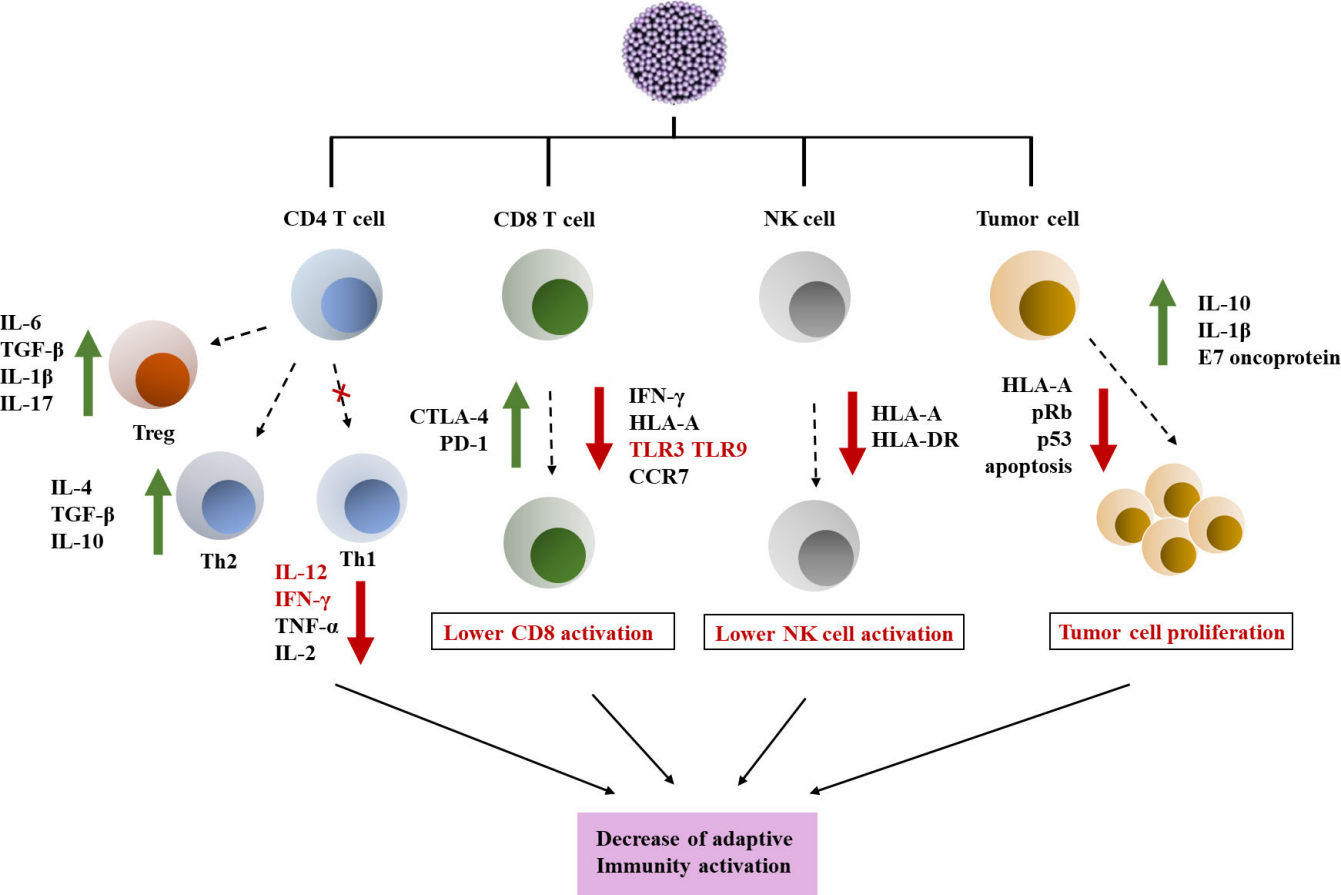
Immune escape induced by HPV in tumor microenvironment. HPV has a certain interfering effect on CD4/CD8 lymphocyte response and NK cells, and affects tumor cell proliferation. (1) HPV can interfere with the expression of TLR and immune signaling pathways. HPV can downregulate Th1 response by reducing pro-inflammatory cytokines. In addition, Th2 and Treg responses are stimulated by the virus. (2) HPV infection can affect the activity of CD8 T cells and also affect antigen presentation, resulting in reduced expression of TLR and CCR7 on the cell membrane surface. In addition, it also showed upregulation of the expression of CTLA-4 and PD-1 inhibitor molecules (6). (3) HPV has a toxic effect on NK cells, leading to downregulation of HLA and weakening of NK cell activity. (4) HPV promotes changes in cell cycle regulatory genes such as p53 and pRb, leading to tumor cell proliferation and promoting the establishment of tumor chemical microenvironment (7).

Avoiding immune tumor monitoring is an established characteristic of cancer (5). In HPV-related tumors, tumor viruses are responsible for immune evasion (**Figure 1**) (8). The persistence of HPV is a critical incident in the natural history of HPV infection and HPV related cancer advancement. Studies have shown that adaptive responses to HPV infection may take several months to develop (9). The constituent of the innate immunity system may has a marginal usefulness in the outcome of HPV infection (10). HPV can eliminate the original process of the innate immune system, which includes the synthesis and secretion of cytokine and Toll-like Receptor (TLR) signaling, thereby impairing the immune response to foreign substances (7). TLR and cytokines play a crucial role in immune defense resist HPV infection and tumor cells. TLR is responsible for identifying conserved pathogen associated molecular patterns (PAMPs), regulating endogenous ligands in the body, and promoting protein cascade reactions to develop immune responses (11). In addition, TLR can control gene expression, which is crucial for creating an appropriate tumor microenvironment, whether for immune regulation and surveillance. TLR is also participated in the nosogenesis of various diseases outside cancer, for example inflammatory diseases, autoimmune diseases and related infectious diseases (12). Therefore, TLR is a key target in immunotherapy research for preventing or treating cancer, and the detection of HPV proteins by TLR agonists has become a promising research field. Actually, they have been used in various drugs for cancer treatment, and several important studies have reported significant intrinsic value in immunotherapy that regulates TLR and cytokines (13, 14).

Based on these studies, this review introduces the HPV virus and HPV related diseases. We discussed the understanding of TLR and its function in HPV infection at present, the changes in TLR expression and signal transduction in skin or mucosal lesions induced by HPV, and immunotherapy methods for HPV related diseases based on TLR agonists. And emphasize the immune mechanism of TLR in the regression of HPV related cancer infection and virus immune evasion activity. This will provide insights into methods that support the treatment of HPV related diseases with TLR agonists.

## 2 Human papilloma virus

**Figure 2:**
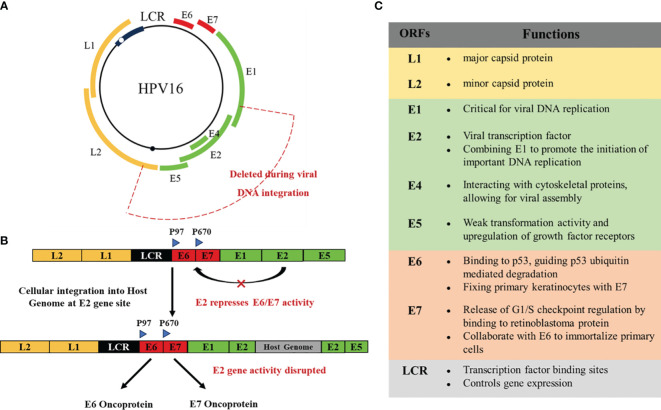
**(A)** Genomic organization of HPV16. **(B)** In the process of developing toward a malignant phenotype, the HPV DNA genome is usually integrated into the host cell genome. The breakpoints generated by integration can disrupt the virus regions encoding E2, E4, E5, and L2. E6, E7, and LCR are usually preserved, leading to the development of cancer. **(C)** The functions of the various structures of HPV.

Papillomavirus is a small circular double stranded DNA virus with a genome of about 8000 bp. It can infect squamous epithelium (or cells with squamous maturation potential) and induce proliferative lesions. These viruses have species specificity and are also highly tissue specific, making them susceptible to infection on the surface of the skin or internal squamous mucosa (15). So far, researchers have identified approximately 130 or more types of HPVs, mainly obtained by sequencing the genes encoding capsid protein L1 (16). The HPV genome consists of three domains: about 1 kb noncoding upstream regulatory region (URR), the early region with open reading frame (ORF) E1, E2, E4, E5, E6 and E7, and the late region which can encode L1 and L2 proteins, which are part of the viral capsid (10). Among them, viral DNA replication requires E1, E2, E4, and E5 proteins. Cancer proteins E6 and E7 cotransform infected cells, while L1 and L2 proteins play an important role in producing HPV viral particles and virus-like particles (VLPs) (**Figure 2**) (17, 18). VLPs are large particles assembled from one or several structural proteins of multiple viruses, which do not contain viral nucleic acids and cannot replicate autonomously. They have a similar overall structure to viral particles.

Only a few high-risk HPV infected cervical pathology will develop to cervical cancer (19). E1 and E2 are the initially expressed proteins that monitor the replication and transcription of viral (20). E2 is a transcription regulatory factor that early expresses HPV genes. Once E2 binds to the four E2 binding domains in LCR, it triggers its control over the transcription levels of oncogenic genes in E6 and E7 viruses (10). In malignant tumors, HPV can be integrated into the host genome. Therefore, the integration of HPV genome may be the main evidence for cancer. The HPV integron may appear in multiple non recurrent amplification regions and bilateral deletion regions. There is a close relationship between HPV insertion breakpoint and genome structural variation, including chromosomal translocation, deletion, inversion and intrachromosomal rearrangement, which ultimately leads to genome instability (3). During this process, breaks often occur at any location within the E1-E2 region of HPV DNA. When E2 breaks, E6 and E7 begin to be upregulated, promoting cervical transformation (21–23).

**Figure 3:**
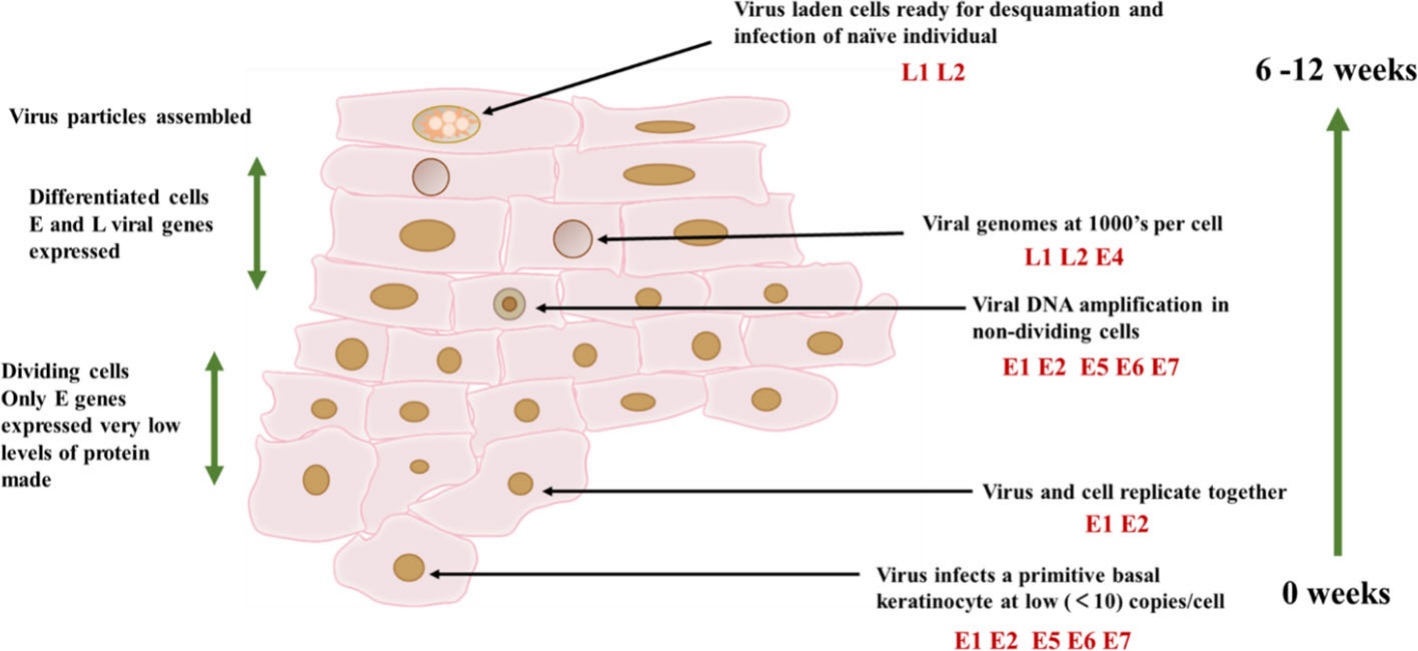
HPV16 infection cycle. The infection cycle of HPV depends on the differentiation process of keratinocytes. The virus can infect keratin in the basal layer of the epithelium, thereby forming cells. And cells can only form in the final differentiated keratin. At this point, the virus particles and virus capsid proteins are assembled together.

HPV can infect primitive basal keratinocyte and may target stem cells, but viral assembly and high-level expression of viral proteins only occur in the upper layer of the spinous layer and in the granules of the squamous epithelium (24) (**Figure 3**). When infected keratinocyte enters the differentiation compartment and exit the cell cycle, the expression of viral genes is largely up-regulated, and viral DNA replication occurs. The number of viral copies can be expanded to at least 1000 copies. Upregulation of early gene E6 and E7, as well as late gene expression (25). HPV infected keratinocyte will also activate a powerful antiviral defense system, namely type 1 IFN. Research has found that HPV16 alters the expression of three sets of genes, namely IFN responsive genes and nuclear factor κB (NF-κB) stimulating genes and cell cycle regulator gene (26, 27). The immune response plays a crucial role in clearing most infections, but some infections cannot be eliminated and can become additional risk factors after years of accumulation (28). However, in order to evade the immune response, E6 and E7 proteins play the most crucial role. The viral mechanisms of immune escape include regulating the expression of cytokines and chemical attractants, altering antigen presentation, and downregulating the IFN pathway and adhesion molecules (29). Avoiding immune response is crucial in the successful infection of HPV (**Figure 1**). HPV has a certain interfering effect on CD4/CD8 lymphocyte response and NK cells, and affects tumor cell proliferation.

## 3 Toll-like receptors

The continuous infection of high-risk HPV can lead to the development of many skin and genital cancer lesions (30). The escape of the innate immune system is usually the main characteristic of the persistence of HPV and the development of tumors. The activation and expression changes of TLR may have a certain effect on controlling HPV infection and may promote lesions and the progression of tumors. HPV can infect primitive basal keratinocyte. The epidermal keratinocyte have been proved to express TLR1, TLR2, TLR3, TLR4, TLR5, TLR6 and TLR9, which can help keratinocyte cells and become the first responder of pathogenic microorganisms (31). HPV replicates in the mucosal layer, and 13 different TLRs have been identified to be expressed in the mucosal layer, which may have an impact on HPV in the mucosal layer (32).

TLR is a specialized receptor for detecting pathogen-associated molecular patterns (PAMP) and damage-associated molecular patterns (DAMP). TLR mainly exists in immune (such as antigen-presenting cells (APC), natural killer (NK) and non-immune (such as stromal, epithelial, and cancer) cells (12). TLRs 1, 2, 4, 5, 6, and 10 are present in the plasma membrane, and TLRs 3, 7, 8, and 9 are present on the surface of organelle such as endoplasmic reticulum, lysosome, and endosome (12). TLR consists of extracellular N-terminal and intracellular C-terminal domains. The extracellular domain contains repetitive sequences rich in leucine that are responsible for recognizing PAMP, depending on the TLR subtype. There is a conserved region at the C-end, known as the Toll/IL-1 receptor (TIR) domain, responsible for transduction of signals from adapter molecules.

**Figure 4:**
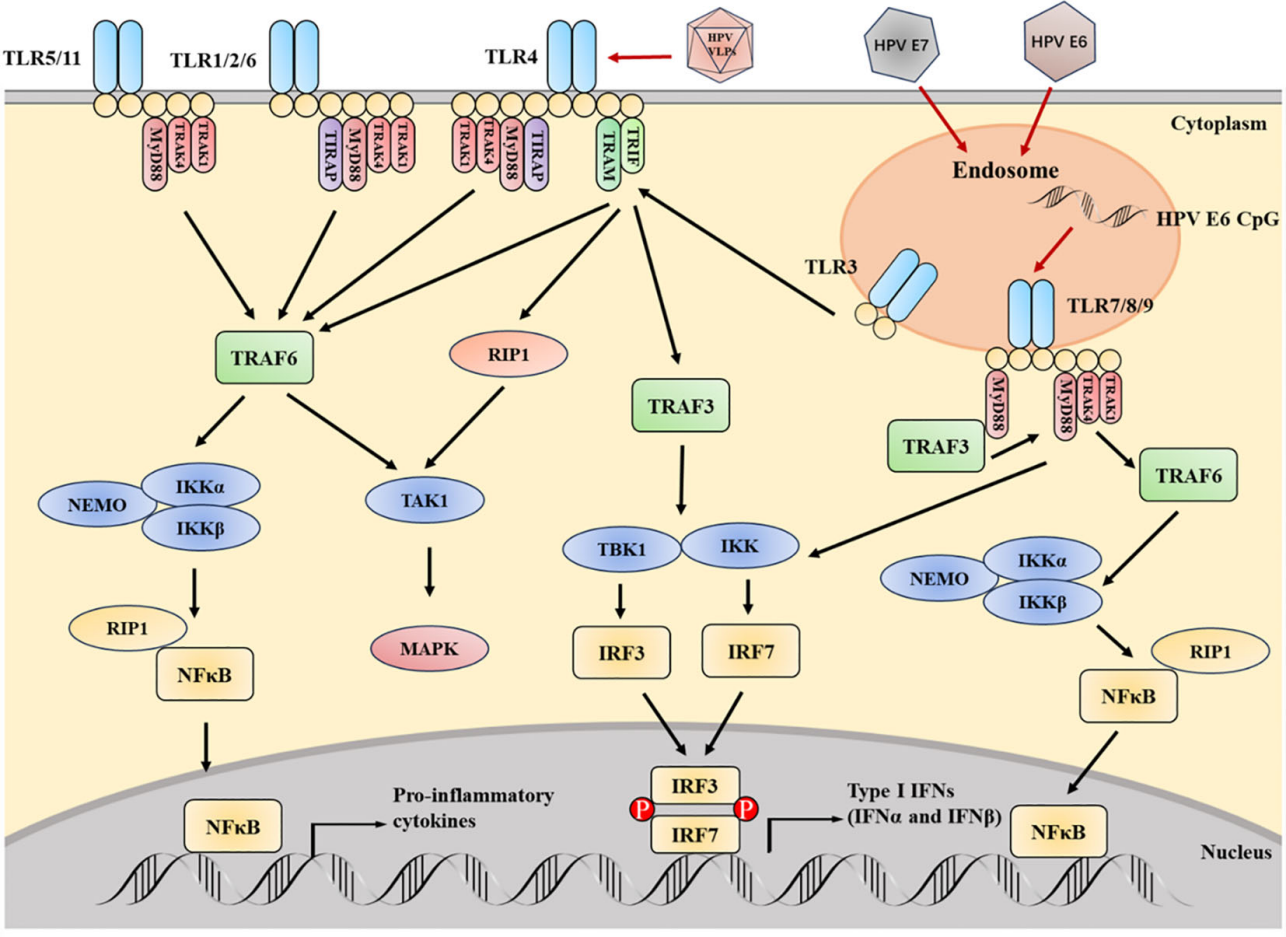
The key TLRs, as well as their signal adapters and downstream media, are crucial for TLR signal transduction. HPV can affect the expression of TLR, which is beneficial for sustained viral infection and even carcinogenesis. The CpG motif from HPV E6 can stimulate TLR9. Infection with HPV E6 and E7 inhibits TLR9 transcription. Contrary to the downregulation of TLR9, the TLR3, 5 and 8 pathways are upregulated during HPV infection. The signal transduction initiated by TLR can activate NF- κB Then activate IRF. The activation of IRF can induce IFN - I, which is crucial for the innate immunity of the virus. HPV L1 VLP can directly activate B cells through TLR4 and MyD88 dependent pathways, thereby producing IgG. In the figure, it should be noted that TLR5 and 11, as well as TLR2 and 6, are independent entities, but their pathways are similar.

The pathways related to TLR signal transduction can be roughly divided into MyD88 dependent and TRIF dependent pathways (**Figure 4**). Except for TLR3, all TLRs are mediated pass through the MyD88 dependent pathway (33). The MyD88 dependent pathway mediated by TLR7 and TLR9 induces type I IFN in cascade, and also involves TRAF3 (34, 35) and IRF7 (36, 37). After phosphorylation, transfer of IRF7 to the nucleus and induction of the expression of IFN-α and IFN-β (38). The induction of inflammatory cytokines and type I IFN is carried out through the TRIF dependent pathway initiated by TLR3 and TLR4. The TRIF dependent pathway activates NF-κB through two independent pathways. The structural domain located at the N-terminus of TRIF can interact with TRAF6, thereby activating NF- κ B. The structural domain located at the C-end of TRIF can interact with RIP1, resulting in the activation of TAK1 (39, 40). IRF7 is an IFN-α Induced main regulatory factor, while IRF3 is responsible for producing IFN-β Key transcription factors required. TLR3 adopts the TRIF dependent pathway, phosphorylates through IRF3, and is activated by IKK related kinases TBK1 and IKKϵ to generate IFN-β (41, 42). They induce NF-κB pathway prevents the activation of immune response or enhances inflammation, which is related to malignant transformation of tumors. Cancer cells may also express these molecules to change immune response and maintain malignant progress (11, 43). Typically, TLR is related to clearing viral infections, preventing pathogen invasion, and preventing cancer, which is crucial for inducing and connecting innate and adaptive responses (including Th1 and toxic cell mediated subtypes) (11). In addition, TLR can recognize some host endogenous ligands and play a critical role in tissue repair and *in vivo* balance (44). TLR2 and TLR1 are involved in many Viral protein components. TLR7, TLR8 and TLR9 have high sequence homology and can bind a series of DNA and RNA molecules. single stranded RNA rich in viral uridine and guanosine (45, 46).

The expression of TLR4 and 9 changes during HPV infection. In studies on the occurrence of cervical cancer, it has been found that TLR4 and 9 levels are crucial for initiating innate immune responses owing to the toxic effects of target cells and the induction of cytokine synthesis. Therefore, lower levels of TLR4 and 9 are typically associated with worsening cancer progression and poor prognosis (47, 48). Overexpression of TLR4 was also found in precancerous and malignant cervical cancer specimens, as well as in the human cervical cancer line (HeLa) (49, 50). It produces TGF-β, IL-6 and other immune regulatory cytokines are associated with cancer cell proliferation and immune suppression (43). TLR9 also shows overexpression in high-grade cervical lesions or cancer (47, 51). The regulation of TLR9 levels may be owing to the HPV16 E7 oncoprotein avidity on the TLR9 promoter interfering with NF-κB. Inhibition of TLR9 expression can interfere with the synthesis of IL-6, IL-8, etc. (52). Due to the deficiency of interferon caused by TLR9 inhibition, the viral transcription and replication process may be interfered with (48). Recently, TLRs have been shown to activate the nitric oxide signaling pathway, leading to the development of cervical cancer (53).

## 4 TLR and HPV-associated cancers

### 4.1 Cervical carcinoma

**Figure 5:**
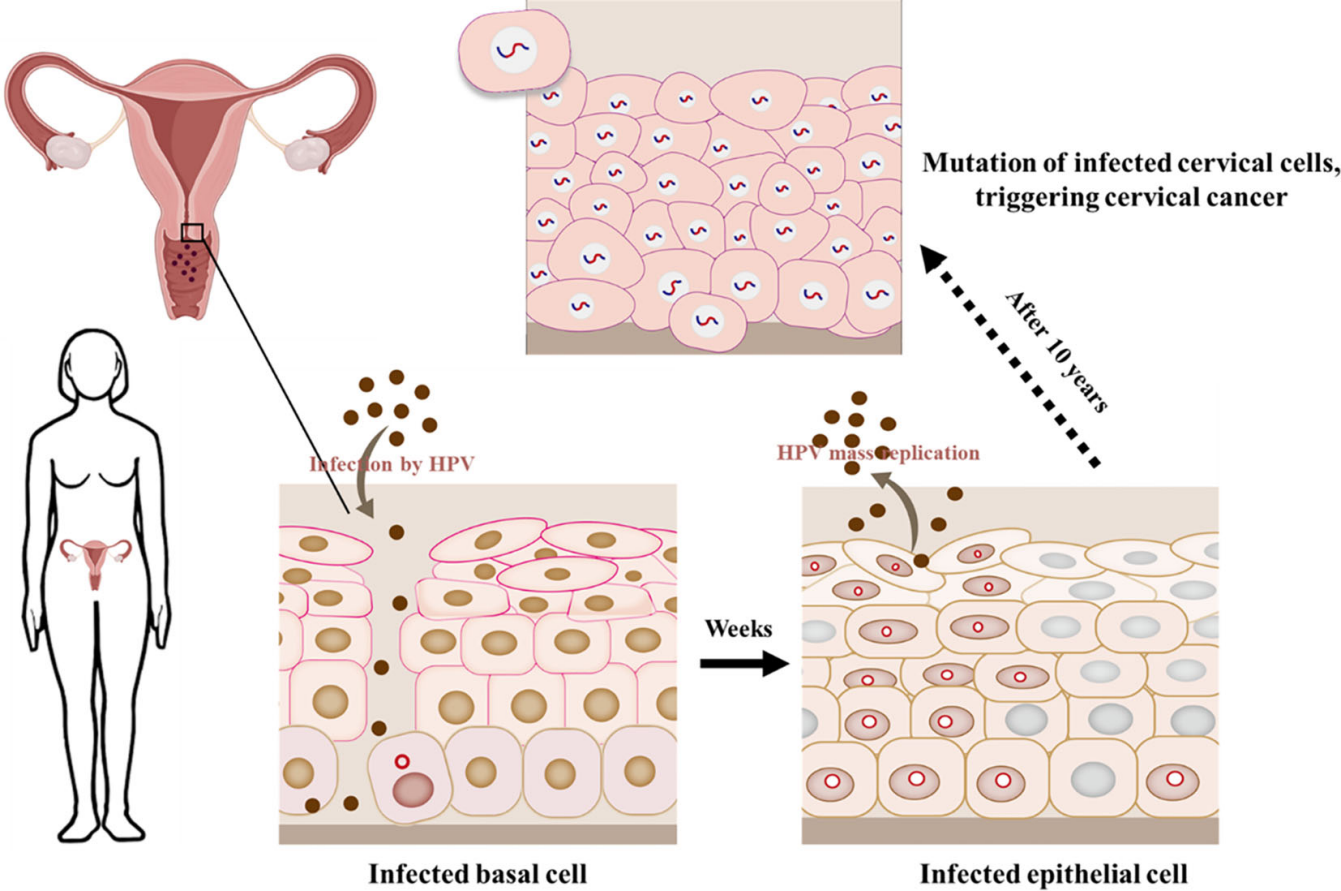
HPV infects epithelial cells in the cervical mucosa. HPV DNA integrates into the cellular genome when causing cancer.

Cervical cancer is a common cancer among women in the world, containing HPV DNA sequences from high-risk genitalia HPVs (54). HPV 16 and HPV 18 are important participants, with HPV 18 occurring in 7% -20% of cases, while HPV 16 can occur in 50% -70% of cases (54). A series of intraepithelial abnormalities occur before cervical cancer and other genital cancers. In the cervix, these forms range from high grade CIN (cervical intraepithelial neoplasia) 3, moderate CIN2, and low grade CIN1 spectra. High grade CIN3 lesion is the specialized progenitor lesions of cervical cancer, with approximately 90% of CIN3s containing high-risk HPV DNA sequences, and HPV 16 being the main participant (27). Persistent infection of high-risk HPV will become the essential hazard factor in the evolution of cervical precancerous lesions. Most infected women have to advantage eliminated the virus, but only a few will develop cancer. In some cases, the adaptive and innate immune responses induced by HPV cannot clear up the virus, reaching to persistence infection and improving the possibility of CIN and cancer procession (55). HPV initially infects basal cells, but after several weeks, the virus replicates extensively in epithelial mucosa and continues to infect, ultimately leading to the development of cancer (**Figure 5**).

The types of HPV that are prone to cancer have affinity with immature squamous cells in the infected cervix. This area is called the transformation region, which happens to where the squamous epithelium outside the cervix transforms into columnar cells in the cervical canal (56). HPV18 E7 can reduce the expression of IRF-7 in cervical tissue of HPV18 E6/E7 transgenic mouse (57). HPV18 E6 can suppress the phosphorylation of Tyk2 kinase STAT1 and STAT2 molecules participated in IFN signal transduction in cervical cancer cell lines (58). Interestingly, when E7 transcript levels are low, HPV16 positive patients with precancerous lesions are susceptible to IFN- α more effective response to treatment (10). HPV infection plays a crucial role in the developing process of cervical cancer, including the interference of keratinocyte reaction, the reduction of LC (Langerhans cell) number and the down-regulation of LC activation markers. The epithelium near the lesion may have an inflammatory microenvironment (59). A study analyzed the changes in gene expression in cervical cancer patients and observed a steady increase in gene expression related to antiviral response and cell cycle, proving that these processes are important for tumor progression (60). Studies have shown that HPV16 E6 and E7 proteins inhibit NF-κB in cultured cervical epithelial cells activity, and this process is helpful to malignant transformation. However, this effect is particularly related to E7 protein and varies between cell cultures in different confluence and proliferation states (61). For example, HPV16 E6 increases NF-κB in unfused and untreated cells activity (61). Some research results indicate that chromosome pairs associated with the induction of antiviral response genes may undergo deletions. However, this chromosomal deletion is a key event in cervical keratinocytes containing HPV16 integration, indicating that the loss of inhibitory extension is an committed step in the development of cervical cancer (27). IFN-β can significantly accelerate the transition from free body to integrated HPV16 in naturally infected keratinocyte, and clear the epigenetic through a non-cytolytic mechanism. Integrated HPV16 can exist for a long time in a small number of cells in polyclonal populations, without selective growth advantage, until it loses its surface layer with E2 depletion and E6/E7 inhibition (27). Some studies have observed that compared with normal Keratinocyte, 80% of differentially expressed genes are down regulated in cervical cancer cell lines (62). Significant changes were detected in both TLR binding genes and molecules related to antiviral immune response, NF-κB activation and MAPK pathway, particularly in the HMGB1-TLR4 signaling axis (62). Analysis of its function also showed that the expression of HMGB1 has a certain effect on the proliferation of cervical cancer cell lines, and also has tumorigenic potential (62).

### 4.2 Skin cancer

Non-Melanoma skin cancer (NMSC) is a primary skin tumor including skin lymphoma, adnexal tumor, Kaposi’s sarcoma, Merkel cell cancer and other infrequent diseases, but it primarily includes two common subtypes of squamous cell carcinoma (SCC) and basal-cell carcinoma (BCCs) (63, 64). HPV is related to several NMSCs (65). Research has found that HPV DNA is present in NMSCs of 30–50% of immunoactivity patients, while HPV DNA is present in lesions of up to 90% of immunosuppressed patients (66, 67). The study found that there was a negative correlation between the malignancy of the lesion and the viral load, which indicates the possible escape mechanism of carcinogenesis (66, 68). β Type HPV (HPV 5, HPV 8, and HPV 9) is an omnipresent virus that can cause widespread infection. During the differentiation of keratinocyte, E6 of HPV 8 can inhibit the activation of NOTCH by inhibiting the RBPJ/AML1 transcriptional activator complex. NOTCH inhibition helps β- HPV replication and viral carcinogenesis deepen the connection between E6 protein and carcinogenicity (69, 70).

HPV infection and ultraviolet radiation can both lead to skin cancer. The damage of ultraviolet radiation to DNA repair activity and the ubiquitous skin HPV type increases the risk of NMSC (65, 71). It can be seen in Genetically modified animal with immune activity and immunosuppression β HPV and ultraviolet rays have a common carcinogenicity. Ultraviolet irradiation of HPV infected primary keratin cells can enhance the promoter activity of HPV 5 and HPV 8, and inhibit local cell-mediated immunity, while HPV oncoprotein can disrupt the renovate of UV dependent DNA damage in keratinocyte (71).

Research has shown that some β Type E6 protein can suppress HIPK2 mediated phosphorylation of p53 serine residues, thereby responding to UV damage. In addition, it can also block the trans activation of p53 target genes, such as p21, MDM2, and some proapoptotic genes (66, 72). This is usually combined with the mechanism of delaying DNA repair, such as the elimination of ATR activity or the damage of telomerase/telomere system, which may be explained the effect of β HPV on skin cancer transformation by promoting the accumulation of UV damaged cells (66, 73, 74). Research has shown that HPV may have a certain impact on the production of BCC, and there are 16 and 18 types of HPV in the serum of SCC patients (75). In addition, the research also found that the positive serum of HPV 5 was related to the hazard of squamous cell carcinoma, the persistent positive serum of HPV 15 was weakly associated with basal-cell carcinoma, and the persistence of HPV 6 antibody was weakly associated with squamous cell carcinoma (75). Researches have also shown that other types of NMSC related to HPV include scrotal squamous cell carcinoma, porous carcinoma, etc. (76, 77).

### 4.3 Head and neck cancer

Head and neck squamous cell carcinoma (HNSCC) is the sixth most usual cancer in the world, with HPV infection being the main cause, especially for oropharyngeal squamous cell carcinoma (OPSCC) (78). Due to HPV activity, HNSCC is often considered an immunosuppressive disease and an inflammatory tumor. Therefore, imbalanced cytokine profiles, decreased levels of CD3, CD4, and CD8 T lymphocytes, decreased antigen presentation, impaired NK cell activity, improved expression of inhibitory receptors, such as TIM-3 and PD-1 were observed (79, 80). More and more evidence support a correlation between HPV infection, especially HPV16 infection, and oral squamous cell carcinoma, especially in people under 50 years old (81).

Research has found that TLR2 was up-regulated in nearby malignant keratinocyte and immune cells in oral squamous cell carcinoma (OSCC) microenvironment (82). In another study related to OSCC, the results showed that TLR3 upregulated its mRNA and protein expression levels in the three head and neck cell lines studied. When inducing tumor cell apoptosis *in vivo*, the expression level is upregulated, similarly, when blocking tumor growth, its expression level is also upregulated. In this research, mRNA expression of other TLRs was evaluated and increased expression of TLRs 1, 2, 4, and 6 was observed, while decreased expression of TLRs 5, 7, 8, and 10 was observed (83). Corresponding evaluations have also been conducted on TLR4 and 9 in OSCC, and the results show that their increased expression levels can lead to tumor development (43).

In other studies, TLR5 and 7 have also been found to be related to the HPV status of tumors in OPSCC (84). TLR2 is associated with HNSCC and oral carcinogenesis (85, 86). Another study measuring TLR3 in various HNSCC cell lines also found an increase in TLR3 levels (87). Studies have found that in OPSCC that detect HPV positivity, disease-specific survival rates are low, which may be related to high expression of TLR5, typically exhibiting a decrease in TLR5 immune expression. *In vitro*, can be detected the cascade activation of NF-κβ in HPV positive OPSCC cell lines (88).

These studies indicate that TLR is an important factor in HPV carcinogenesis. The roles of different HPV subtypes and TLRs in related cancers are complex and diverse, and further research is needed to explore their pathological mechanisms. These studies will help to better understand the pathological mechanisms of cancer and provide theoretical basis for the development of related treatment methods.

## 5 Treatment of HPV related cancers

### 5.1 Single and combined use of TLR agonists

Agonists can be used for treatment aimed at disrupting the anti-inflammatory microenvironment produced by E6 or E7 HPV positive cells, thereby resisting the tolerance of HPV oncoprotein production and playing a therapeutic role (10). When treating HPV related cancers such as head and neck cancer and cervical cancer, it has been considered to regulate TLR expression or activity as adjuvants in various kinds of vaccine strategies. TLR agonists are actively being used in research and treatment of solid tumors (89, 90). The purpose is to increase the synthesis of chemokines and cytokines such as TNF-α and IFN. Usually, CTL cells are activated to produce effector responses by activating dendritic and NK cells (91). Motolimod (VTX-2337), a TLR8 agonist, can promote the activation of T cells by inducing the synthesis of cytokines and chemokines and activating Monocyte, NK and dendritic cells to increase anti-tumor activity. Tested for use in the therapy of HNSCC and other tumors. Imiquimod is a TLR7 agonist, a synthetic imidazolium quinoline. It is commonly used for external genital warts (genital wart), superficial basal-cell carcinoma and actinic keratosis (92). The immune activation mechanism of imidazoloquinoline is through recognition of TLR7 or TLR8 and activation of the MyD88 dependence signaling pathway (93). Imiquimod can stimulate Th1 response by inducing DC migration and maturation, migration of Langerhans cell to draining lymph nodes, inhibiting myelogenous sexual inhibition cells, and secreting interleukin and cytokines (94). Poly (I: C) is the mimic of viral dsRNA (95). It is also a TLR3 agonist aimed at simulating pathogen invasion and enhancing immune system activation to advance anticancer treatment, and has certain therapeutic effects in the therapy of cervical cancer and squamous cell carcinoma (96). In addition, TLR3 activation in HCC *in vivo* model can up regulate chemokines in tumor and increase the activation of tumor infiltrating immune cells. These studies conducted *in vitro* indicate that apoptosis caused by TLR3 can be used to explain why patients expressing TLR3 have a well prognosis and the corresponding mechanisms (96). Poly (I: C) also upregulated the expression of class I MHC molecules on the surface of HeLa (97). During viral infection, the enhancement of viral specific CTL presentation by intracellular antigens can be achieved through upregulation of class I MHC molecule expression, which is essential for preventing pathogen transmission within cells and identifying infected cells (98). Besides poly (I: C) induces higher levels of proinflammatory cytokine secretion, such as CXCL-10, IL-6 and IFN-I, tumor cell MHC I expression and *in vitro* monocyte activation. Even if TLR signal transmission is blocked on host cells, tumor growth will be damaged *in vivo* and *in vitro* (99). Researches have applied poly (I: C) to CAL-27, a kind of HPV negative oral cell, demonstrating that poly (I: C) may be an effective potential therapeutic strategy for increasing prognosis and radiation response in HPV negative OSCC (100). Due to the limited toxicity of Poly (I: C), several improved versions have been developed, such as PolyI: PolyC12U17 and Poly (IC: LC) 1, to reduce toxicity (101, 102). In another study, the effects of poly (I: C) and imiquimod on cell death *in vivo* and *in vitro* HNSCC models were estimated (14). It was found that both agonists can lead to increase the mortality rate of tumor cells *in vivo* and *in vitro*. Molecule 852A, also named as S-32865, is a novel immune response modulator (IRM) that is also associated with the imidazole quinoline molecule imiquimod and can serve as a kind of TLR7 agonist. 852A conducted the first human phase I trial in patients with refractory solid organ tumors (103). Testing the subcutaneous administration of 852A in refractory cervical malignancies. 852A shows sustained tolerance in some patients. However, the clinical benefits are not significant and have cardiotoxicity (93). CV8102 is a novel synthetic non coding long chain RNA molecule with a polymer carrier that can induce balanced, support strong anti-tumor activity and persistent immune responses in preclinical models (104). Observing profound anti-tumor effects. Adjuvants act locally at the injection site and do not induce systemic cytokine release. Direct comparison with Poly (I: C) shows that CV8102 has excellent efficacy in enhancing antigen-specific multifunctional CD8 (+) T cell responses and mediating anti-tumor responses induced by HPV 7 E1 protein derived peptides in homologous TC-16 tumors (a mouse model of human HPV-induced cervical cancer) (104). Phase II clinical trials using CV8102 alone in the treatment of HNSCC are currently being recruited (105). 3M-002, a kind of TLR-8 agonist or resiquimod, a kind of TLR-8 and 7 agonists, combined with viral particles VLP-L1-L2-E7 or VLP-L1-L2 can activate LC and induce specific CD8+T cell responses (98). 3M-002 and imiquimod, together with HPV11 E7 epitope, can up regulate HLA-DR, CD40, CD80, CD83, CD86, cytokines such as IFN-γ and IL-12 in monocyte derived dendritic cells (mdDC) in human HPV infected tissues-γ. It can also promote specific cytotoxic reactions of T lymphocytes (106). Recombinant lipoproteins with intrinsic TLR2 agonist activity contain bacterial lipid fractions (rlipo E7m) and mutant forms of E7 (E7m), which have been shown to induce strong CTL (cytotoxic T lymphocyte) responses targeting small tumors. When combined with CpG (TLR9 agonist) and rlipo (TLR2 agonist), they can effectively increase tumor infiltration in CTL, and reduce the number of myeloid-derived suppressor cells (MDSC), regulatory T cells (Tregs) and tumor-associated macrophage (TAM) in the tumor microenvironment (107). Many studies have shown satisfactory results, especially when using these agonists simultaneously. Generally speaking, an increase in tumor cell mortality was observed after TLR stimulation (91). In addition, it is reported that the cure rate of the original tumor in mice is very high (107, 108).

### 5.2 Combination therapy of vaccines and TLR agonists

There are several efficient vaccines that can block the infection of HPVs, thereby preventing cervical cancer, anal genital cancer, and head and neck cancer caused by these viruses. However, none of these vaccines can effectively treat HPV induced tumors (109).

In HPV related cancers, several pharmacological substances that alter the activity of these receptors were first tested and used as adjuvants for cervical cancer vaccines. Cervarix is a vaccine which can target HPV 16 and 18. MPL (LPS derived from Salmonella Minnesota) is included in Cervarix’s formula and is a TLR4 agonist (92). MPL can be used to promote to activate the innate response of interferon and synthesis pro-inflammatory cytokine. As a result, it can also trigger adaptive responses (14). CIA05 is a TLR4 agonist. It originates from the Escherichia coli lipopolysaccharide (LPS) mutant and has been shown to have the potentiality as a vaccine adjuvant (110). On the basis of CIA05, we also studied the immune enhancing activity of a novel adjuvant system CIA06 composed of aluminium hydroxide (alum) and CIA05. When it is used with HPV L1 VLP vaccine, the titter of anti HPV L06 VLP IgG antibody in serum, splenocyte interferon IFN-γ secretion and antigen-specific memory B cell response produced the highest immune response. Compared with the immunogenicity of the currently licensed HPV vaccine Cervarix, it is similar to Cervarix in inducing virus neutralizing antibody and serum antigen specific IgG antibody, but more available in inducing the production of memory B cell and splenocyte factor (111). Gardasil is a tetravalent vaccine, which is composed of type 1, type 6, type 11 and type 16 HPV L18 VLP produced in yeast, and uses hydroxy aluminium phosphate and aluminium sulphate as adjuvants (112). Patients with central recurrence of cervical cancer receive at least two intratumoral Gardasil injections and local injection of imiquimod on the tumor surface. The addition of a combination of local TLR7 agonist therapy on the basis of intratumoral vaccination can unexpectedly lead to complete regression of the disease in two patients (113). Due to the potential lack of immunogenicity of peptides themselves, it is widely believed that standard/conventional vaccine adjuvants may not provide the intense inflammatory signals required to provoke specific responses against peptide pointed at treatment tumor vaccines. It has been demonstrated in a study that the synthesis of polyI: C form dsRNA as an effective adjuvant to increase specific anti-tumor immune responses against peptide pointed at vaccines. When PolyI: C binds to the MHC I restrictive minimal peptide epitope originated HPV 16 E7 protein, a PolyI: C/E7 molecular complex is produced. This complex leads to an intense E7 specific CTL response, which in turn leads to significant regression of human cancer tumors previously established in mouse models (114). In order to treat HNSCC, a therapeutic vaccine experimental strategy based on plasmid DNA encoding retroviral like particles (pVLP) has been developed, which combines DNA vaccination and VLP formation for the treatment of TC-1 tumor bearing mice. Research has found that the pVLP strategy is more effective in inducing antigen-specific immune responses and anti-tumor effects than using DNA vaccines alone or VLP. When the therapeutic vaccine of pVLP-E7 is administered in combination with TLR agonists such as CpG-ODN and imiquimod, it can control the growth of advanced tumors and cure 50% of mice, thereby achieving long-term disease-free survival (115).

### 5.3 Combination therapy of checkpoint inhibitor monoclonal antibodies and TLR agonists

At present, monoclonal antibodies against PD-1 and TIM-3, such as pembrolizumab and nivolumab, have been cited for research and used to treat solid tumors (116). The other side of the shield, the combination of these monoclonal antibodies and TLR agonists in the treatment of HPV related cancers is novel. 1V270 is a TLR7 agonist with a very low molecular weight, coupled with phospholipids, and is an efficient and stable immune activator (117–119). The lipid part of 1V270 can prevent unnecessary systemic cytokine storm and improve the safety after local administration (120). SD-101 is a TLR9 agonist, which is a nucleotide thiophosphate oligodeoxynucleotide with multiple immune stimulating CpG motifs (CpG-ODN) (121, 122). Research has found that I.t. therapy using SD-101 or 1V270 combined with anti PD-1 antibodies can activate TAMS and guidance tumor specific adaptive immune responses, thereby inhibiting primary tumor growth and preventing metastasis in the HNSCC model (123). Therapeutic pathways for TLR7 or TLR9 agonists to enhance the curative effect of PD-1 antagonist in HNSCC (123). Cetuximab is an anti EGFR monoclonal antibody. If used in combination with the TLR8 agonist Motolimod, research has found that it can lead to increased NK cell-mediated cancer cell lysis, and EGFR specific CD8 T cell DC cross initiation was enhanced (38). Other probabilities of Motolimod are being tested at present, for example, combining it with Cetuximab/fluorouracil/cisplatin or carboplatin/Nivolumab/Cetuximab and Ghana Vuliumab (105). PolyICLC is an improved version of poly (I: C). There are studies currently recruiting participants to assess the curative effect of polyICLC in combination with PD-L1 antibody durvalumab and CTLA-4 antibody tremelimumab (14). The combination of CV8102 and PD-1 monoclonal antibody for the therapy of SCC is currently being recruited for its Phase II clinical trial (105). Therefore, the combination of different immunotherapy methods shows better therapeutic effects. **Table 1** summarizes the treatment methods for TLR agonists and HPV related diseases mentioned above.

**Table 1:** TLR agonists associated with the treatment of HPV related diseases.

Target	Agonist	Disease/model	Therapeutic method	stage	References
TLR7	Imiquimod (852A)	Superficial Basal-cell carcinoma and external genital warts	As single agent	Phase II	(92)
		Cervical cancer	Combined with Gardasil	Phase I/II	(103)
		Cervical cancer	As single agent	Phase II	(93)
TLR7	1V270	HNSCC model	Combined with anti PD-1 antibody	*In vitro*/*in vivo*	(123)
TLR8	Motolimod (VTX-2337)	HNSCC	As single agent or combined with nivolumab or combined with nivolumab or combined with cetuximab and nivolumab	Phase I/II	(105)
TLR8	3M002	Human immune cells isolated from peripheral blood lymphocyte B (PBL)	Combined with VLP-L1- L2/VLP-L1-L2-E7	*In vitro*/*in vivo*	(98)
TLR7/TLR8	Resiquimod	Human immune cells isolated from peripheral bloodlymphocyte (PBL)	Combined with VLP-L1- L2/VLP-L1-L2-E7	*In vitro*/*in vivo*	(98)
TLR7/TLR8	CV8102	HNSCC, SCC	As single agent or combined with standard of care PD-1 blockage	Phase I/II	(105)
TLR3	Poly (I: C)	Cervical cancer, SCC, HCC	As single agent	Phase I/II	(96)
		HNSCC	Combined with Miquimod	*In vitro*/*in vivo*	(14)
TLR3	polyICLC	HNSCC	Combined with tremelimumab and durvalumab	Phase I/II	(14)
TLR9	CpGODN	Mouse model	Combined with E7 recombinant protein	*In vitro*/*in vivo*	(107)
TLR9	SD-101	HNSCC model	Combined with anti PD-1 antibody	*In vitro*/*in vivo*	(123)
TLR4	MPL	Cervical cancer	Included in Cervarix	approved	(14)
TLR4	CIA05	Cervical cancer	Combined with HPV L1 VLP vaccine	Phase I/II	(110)
TLR4	CIA06	Cervical cancer	Combined with HPV L1 VLP vaccine	Phase I/II	(111)

In short, the treatment of cancer is a multifaceted and multidisciplinary process. Combining the tumor microenvironment with personalized combination therapy using TLR agonists and various treatment methods will become an effective method for clinical treatment of cancer.

## 6 Future prospects

HPV infection plays a crucial role in sexually transmitted diseases, common skin diseases, and some cancers in the world. With the increasing accessibility of cancer screening and early cervical diseases, the progress of cervical cancer screening and early cervical diseases is also continuing. This progress has led to a decline in the incidence rate and mortality of cancer. However, for patients with invasive, advanced, or recurrent cervical cancer, traditional treatments including systemic chemotherapy, surgery, and combined local radiotherapy are still largely ineffective. Although progress has been made in the study of host HPV interaction patterns, substantial benefits have not yet been achieved for patients. Cervical cancer vaccines are one of the effective measures to prevent cervical cancer. However, there is controversy over the age and frequency of vaccination, and it does not cover all HPV subtypes (124, 125). Therefore, it is necessary to further develop a more cost-effective and effective treatment method. The current research mainly focuses on the relationship between HPV infection and cervical cancer, while research on other factors is still insufficient. Although molecular classification and personalized biomarkers for any HPV variant have been established (126), further research is needed to understand the molecular subtypes of cervical lesions and continue to develop drugs for better diagnosis and treatment. Therefore, emerging treatment tools, such as targeted immunotherapy, therapeutic vaccines, and TLR agonist therapy, provide new hope for solving the problem of HPV carcinogenesis.

The immune response is crucial in the resolution and progression of HPV related cancer diseases. Observing that host immune responses are beneficial to patient health in all kinds of ways, as these characteristics occur during the natural regression of infection. These new immunological methods have opened up new perspectives for diagnosis, especially for cancer treatment. Nowadays, the utilization of them seems to be a very prospective immunotherapy strategy. Some monoclonal antibodies can block the activity of immune checkpoint molecules such as PD-1 and CTLA-4. In the pathogenesis of tumors, the activation of these molecules can trigger signaling pathways, thereby blocking the function of CD8 and CD4 cells. In addition, inducing other immune evasion activities provides a suitable environment for the continuation of infection and tumor development. High expression of CTLA-4 in HPV related cancers (79). PD-1 is closely related to immune escape in HPV related cancers, and is associated with decreased disease survival caused by decreased CTL activity (127). Therefore, inhibiting these activities can be considered a good therapeutic target. The combination of anti-vascular endothelial growth factor antibody bevacizumab and chemotherapy extended the survival period of patients with advanced cervical cancer by 3.7 months (128). Oral tyrosine kinase inhibitor lapatinib can enhance the sensitivity of cervical cancer cells to paclitaxel, reduce tumor microvascular density, and thereby inhibit the formation of neointima in tumor tissue (129). T cell immune checkpoint inhibitors targeting the PD-1-PD-L1 axis have been shown to be tolerant in patients with recurrent or metastatic cervical squamous cell carcinoma who are PD-L1 positive (130). These therapies tend to minimize damage to normal tissues by targeting specific molecules. However, challenges such as individual variability and drug safety still exist.

TLRs are considered to be a protein family, which are involved in inducing innate immune response, fighting against bacteria and viruses. Nowadays, research on the relationship between innate immunity and cancer has attracted widespread attention, and this study actually provides key information for developing cancer treatment strategies. Research suggests that the TLR expression profile may be one of the guidelines for diagnosing specific cancers. By using TLR agonists or antagonists in combination with antigens isolated from tumors, we can expect an increase in vaccine efficacy and evoke specific innate immunity against cancer. In addition, it has been proven that TLR mediated signaling induces tumor growth, and in some cancer models, tumor cells have been observed to escape from the host immune system. The combination of TLR and monoclonal antibodies in immunotherapy is a hot research topic. The combination of different immunotherapy methods, such as the combination of different TLR agonists, checkpoint inhibitor monoclonal antibodies, and different agonists, has shown increased beneficial effects in preclinical and clinical trials. Currently, the combination with TLR agonists is novel for HPV related cancers. There are only two studies: NCT02643303 and NCT02124850 (14). Currently, patients are being recruited to test the combination of poly (I: C), tremelimumab, and durvalumab, as well as VTX-2337 plus cetuximab or VTX-2337 plus cetuximab plus gaburizumab (105). There is currently little research on the joint evaluation of checkpoint inhibitors and cytokines in HPV related cancers, with only reports on other solid tumors unrelated to HPV (14). However, research on TLRs is still in its early stages, and researchers need to explore the more detailed functions of each TLR in different types of cancer in order to develop treatment strategies using the TLR pathway. In addition, HPV related tumors require a good state of immunosuppressive factors to promote cancer development, such as increased activity of PD-1 and Treg and inhibition of NK cells. Therefore, studying this evasion mechanism and combining TLR agonists with various cytokines can provide new therapeutic perspectives.

In summary, the treatment of cervical cancer with TLR agonists or inhibitors is a very interesting topic. Although these drugs have not yet been widely used in clinical practice, existing research data indicates that this method is effective and has great potential. As research on TLR related signaling pathways continues, it is expected that this treatment method will gain wider acceptance and utilization.

## Author contributions

S-LD: Supervision, Writing – original draft, Writing – review & editing. S-YQ: Formal analysis, Methodology, Writing – original draft, Writing – review & editing. M-MY: Data curation, Methodology, Writing – original draft, Writing – review & editing. C-YL: Visualization, Writing – review & editing. KY: Conceptualization, Funding acquisition, Project administration, Resources, Writing – original draft, Writing – review & editing.
